# Time trends in infectious and chronic disease consultations in Dakar, Senegal: Impact of COVID-19 sanitary measures

**DOI:** 10.7189/jogh.13.06007

**Published:** 2023-03-31

**Authors:** Massamba Diop, Elisabeth LP Sattler, Audrey Geoffroy, Awa Ba Diop, Ibrahima Bara Diop, Camille Lassale, Crystal Cene, Anouk Asselin, Xavier Jouven, Bamba Gaye

**Affiliations:** 1 The African Research Network Noncommunicable diseases (ARNCD), Fann University Hospital Cardiology Department, Dakar, Senegal; 2 S.O.S. Medecin Senegal, Dakar, Senegal; 3 Department of Clinical and Administrative Pharmacy College of Pharmacy University of Georgia, Athens, Georgia, USA; 4 Department of Nutritional Sciences College of Family and Consumer Sciences University of Georgia, Athens, Georgia, USA; 4 Integrative Epidemiology of Cardiovascular Diseases, Paris Cardiovascular Research Center-INSERM U970 (PARCC), Paris, France; 5 Laboratoire de physiologie et d'explorations fonctionnelles, université Alioune Diop de Bambey; 6 Cardiology Department, University Hospital of Fann, Dakar, Senegal; 7 Cardiovascular Risk and Nutrition group, Hospital del Mar Research Institute, Barcelona, Spain; 8 CIBER of Physiopathology of Obesity and Nutrition (CIBEROBN), Institute of Health Carlos III, Madrid, Spain; 9 Department of Medicine, Division of General Internal Medicine, University of North Carolina at Chapel Hill, Chapel Hill School of Medicine, North Carolina, USA; 10 Department of Medicine, University of California, San Diego Health, San Diego, California, USA; 11 School of Medicine, University of California, San Diego, California, USA; 12 Cardiology Department, Georges-Pompidou European Hospital, Paris, France

## Abstract

**Background:**

The impact of COVID-19 sanitary measures on the time trends in infectious and chronic disease consultations in Sub-Saharan Africa remains unknown.

**Methods:**

We conducted a cohort study on all emergency medical consultations from January 2016 to July 2020, from SOS Medecins in Dakar, Senegal. The consultation records provided basic demographic information such as age, ethnicity (Senegalese or Caucasian), and sex as well as the principal diagnosis using an ICD-10 classification (“infectious”, “chronic”, and “other”). We first investigated how the pattern in emergency consultation differed from March to July 2020 compared to previous years. Then, we examined any potential racial/ethnic disparities in COVID-19 consultation.

**Results:**

We obtained data on emergency medical consultations from 53 583 patients of all ethnic origins. The mean age of patients was 37.0 (standard deviation (SD) = 25.2) and 30.3 (SD = 21.7) in 2016-2019 and 45.5 (SD = 24.7) and 39.5 (SD = 23.3) in 2020 for Senegalese and Caucasian patients, respectively. The type of consultations between January and July were similar from 2016 to 2019; however, in 2020, there was a drop in the number of infectious disease consultations, particularly from April to May 2020, when sanitary measures for COVID-19 were applied (average of 366.5 and 358.2 in 2016-1019 and 133.0 and 125.0 in 2020). The prevalence of chronic conditions remained steady during the same period (average of 381.0 and 394.7 in 2016-2019 and 373.0 and 367.0 in 2020). In a multivariate analysis adjusted for age and sex, infectious disease consultations were significantly more likely to occur in 2016-2019 compared to 2020 (2016 odds ratio (OR) = 2.39, 2017 OR = 2.74, 2018 OR = 2.39, 2019 OR = 2.01). Furthermore, the trend in the number of infectious and chronic consultations was similar among Senegalese and Caucasian groups, indicating no disparities among those seeking treatment.

**Conclusions:**

During the implementation of COVID-19 sanitary measures, infectious disease rates dropped as chronic disease rates remained stagnant in Dakar. We observed no racial/ethnic disparities among the infectious and chronic consultations.

The severe acute respiratory syndrome coronavirus 2 (SARS-CoV-2) causing the coronavirus disease 2019 (COVID-19) is an emerging health threat [1]. Europe and the USA appear to not have used optimal strategies until the virus had spread to thousands of people. Specifically, the EU had waited six weeks following the first reported case to implement a COVID-19 response [1]. In the US, there was a large gap in the federal COVID-19 response, allowing the virus to spread to all 50 states two months after the first case was declared [2].

With minimal resources available, sub-Saharan Africa was poorly armed against COVID-19 and was projected to have worse outcomes due to the pandemic compared to developed countries. However, the reverse outcome was observed. Africa has been affected only moderately by COVID-19 until the end of June 2020, after which a new outbreak was observed in most African countries. As of September 17, 2020, the virus had spread to all 54 countries in Africa and has reached a total of about 1 373 926 confirmed cases and 33 255 estimated deaths [3]. Some have hypothesized that the observed differences were due to lower age demographics, higher temperatures, and efficient lockdown implementation [4]. Although sanitary measures in sub-Saharan Africa seemed to help with controlling the number of COVID-19 cases, there is limited research on their effectiveness and their effect on the dynamic of the pandemic and the morbidity and mortality of other prevalent diseases in sub-Saharan Africa. Furthermore, not only has the lack of early interventions led to an unprecedented number of cases and deaths but has also highlighted disparities among different racial/ethnic groups in western countries [5]. In several USA states, COVID-19 mortality was highest among Latinos (187 per 100 000) and African Americans (184 per 100 000) [6]. In the UK, people from Black or mixed backgrounds showed higher COVID-19 infection and mortality [7].

We prospectively evaluated all emergency medical consultations through a major emergency service provider in Dakar, Senegal (“SOS Medecins”), from January 2016 to July 2020. We wanted to assess the COVID-19 pandemic’s impact on emergency consultations in Dakar, Senegal through the type of consultations by time and ethnic profiles. First, we investigated whether sanitary measures have been more effective in Africa. We hypothesized that if sanitary measures were effective in controlling COVID-19, then could they also help reduce the transmission of other infectious diseases. Second, we assessed whether there were disparities between African (Senegalese) and Caucasian subjects observed in Africa like in the US and Europe.

## METHODS

### Data extraction

We extracted data on all emergency medical consultations over a four-and-a-half-year period (from January 1, 2016, to July 31, 2020) using records of SOS Médecins, the largest provider of such services in Senegal. This service was established in 1997, and it maintains rigorous standards, with an ISO9001 international quality certification. SOS Medecins Dakar is operational at all times (24 hours a day, seven days a week) and comprises eight emergency physicians, 11 anesthesiologists, and four cardiologists. It offers mobile emergency consultations and ambulance transport with a physician or paramedic. A standardized protocol is used to document each consultation; details and a provisional diagnosis are entered into the SOS Médecins database by the attending doctor. Three days later, a final diagnosis is ascertained using additional data (phone call to discharged patients and review of the medical records). The SOS Médecins database is a robust source of information on patients who seek emergency care for diverse medical conditions in Dakar.

### Statistical analyses

A dedicated team of physician-researchers obtained data for all consecutive medical consultations handled by SOS Médecins in Dakar. They collected comprehensive demographic information, including age, ethnicity (Senegalese vs Caucasian patients), sex, and instigator of the consultation (family or patient). We then assigned the final principal diagnosis with an ICD-10 classification to three broad groups: 1) “infectious” (respiratory infections, gastroenteritis/diarrhea, otorhinolaryngology/stomato/infectious, and other infectious diseases), 2) “chronic diseases” (cardiovascular disease, rheumatological, neurological, and psychiatric conditions, non-communicable non-infective pulmonary diseases, and gastrointestinal disorders), and 3) others (mainly trauma and accidents/injuries).

We obtained data from patients of all ethnic origins. We first examined and plotted the unadjusted rates of consultations for the three disease groups between January and July from 2016 to 2020. We also stratified the analysis for sex as age groups (<20, 20-45, >45 years of age). We then assessed and compared the unadjusted rates of the two main disease categories “infectious diseases” and “chronic diseases” from April to July (the period corresponding to the COVID-19 outbreak) for each year between 2016 and 2020. After testing for normality, we used the mean and standard deviation (SD) for continuous variables and percentages for categorical variables. We used χ^2^ tests for categorical variables and a two samples *t-*test for continuous variables. For all analyses, we set the type-I error rate to evaluate statistical significance at 0.05.

We used multivariate logistic regression to model the prevalence of infectious disease consultations (compared to chronic disease) across periods. Odds ratios (ORs) and 95% confidence intervals (CIs) were calculated as estimates of the relative risk of examination for infectious disease associated with each period. We performed all statistical analyses using R version 3.6.1.

### Patient and public involvement statement

It was neither possible to involve patients nor the public in the design or the conducting, reporting, or dissemination plans of our research.

### Ethics approval

We obtained ethical approval for this study from the National Ethical Committee of Senegal (number 4864602).

## RESULTS

**Figure 1:**
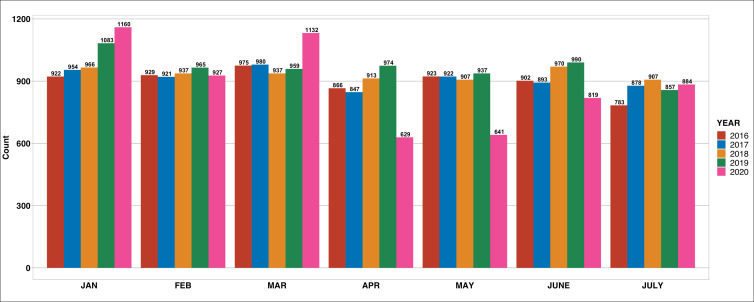
Number of emergency medical consultations by month over a five-year period.

SOS Médecins in Dakar undertook 53 583 medical consultations over the four-and-a-half-year observation period. After excluding consultations with missing and/or incomplete data regarding the sex of the patient (n = 18), age (n = 92), ethnicity (n = 57), and diagnosis for the consultation (n = 119), we obtained complete data for 53 297 consultations. The study flowchart is detailed in Supplemental Figure 1 in the **Online Supplementary Document**. **Figure 1** shows the distribution of emergency medical consultations by month from January to July over the four-and-a-half-year period. Demographic data are shown in **Table 1**. The mean age (in years) of patients was 37.0 (SD = 25.2) for Senegalese and 30.3 (SD = 21.7) for Caucasian patients in the 2016-2019 period, and 45.5 (SD = 24.7) for Senegalese and 39.5 (SD = 23.2) for Caucasian patients in 2020. **Table 1** provides further details on the type of consultations received among the overall population, grouped by infectious and chronic, in five-year bands, and by descriptive data on ICD10 diagnostic categories separately.

**Table 1:** Characteristics of consultations from April to July 2020 compared to the April to July 2016-2019 period in Senegalese vs Caucasian patients*

	April to July 2016-2019				April to July 2020			
	**All**	**Senegalese**	**Caucasian**	**Overall *P***-**value**	**All**	**Senegalese**	**Caucasian**	**Overall *P***-**value**	
	**n = 14 469**	**n = 7281**	**n = 4629**		**n = 2973**	**n = 1825**	**n = 641**		
**Age in years, mean (SD)**	35.2 (24.1)	37.0 (25.2)	30.3 (21.7)	<0.001	44.0 (24.3%)	45.5 (24.7%)	39.5 (23.2%)	<0.001	
**Age as two categories, in years**				<0.001				<0.001	
≤20	4514 (31.2%)	2127 (29.2%)	1786 (38.6%)		534 (18.0%)	302 (16.5%)	158 (24.6%)		
>20	9955 (68.8%)	5154 (70.8%)	2843 (61.4%)		2439 (82.0%)	1523 (83.5%)	483 (75.4%)		
**Age as three categories**				<0.001				<0.001	
≤20	4514 (31.2%)	2127 (29.2%)	1786 (38.6%)		534 (18.0%)	302 (16.5%)	158 (24.6%)		
20-45	5250 (36.3%)	2669 (36.7%)	1582 (34.2%)		1044 (35.1%)	642 (35.2%)	204 (31.8%)		
≥45	4705 (32.5%)	2485 (34.1%)	1261 (27.2%)		1395 (46.9%)	881 (48.3%)	279 (43.5%)		
**Sex**				<0.001				<0.001	
Female	8079 (55.8%)	4348 (59.7%)	2365 (51.1%)		1675 (56.3%)	1075 (58.9%)	324 (50.5%)		
Male	6390 (44.2%)	2933 (40.3%)	2264 (48.9%)		1298 (43.7%)	750 (41.1%)	317 (49.5%)		
**Pathology**				<0.001				.	
All trauma	985 (6.8%)	444 (6.1%)	392 (8.5%)		185 (6.2%)	102 (5.6%)	51 (8.0%)		
Cardiovascular	655 (4.5%)	408 (5.6%)	115 (2.5%)		192 (6.5%)	143 (7.8%)	23 (3.6%)		
Dermatology	356 (2.5%)	112 (1.5%)	177 (3.8%)		56 (1.9%)	19 (1.0%)	30 (4.7%)		
Endocrinology, metabolic tr	162 (1.1%)	125 (1.7%)	11 (0.2%)		46 (1.6%)	35 (1.9%)	6 (0.9%)		
Ent and stomatology (chronic)	381 (2.6%)	193 (2.7%)	122 (2.6%)		89 (3.0%)	56 (3.1%)	17 (2.7%)		
Ent and stomatology (infectious)	1874 (13.0%)	815 (11.2%)	769 (16.6%)		172 (5.8%)	83 (4.5%)	61 (9.5%)		
Haemato-oncology	64 (0.4%)	52 (0.7%)	5 (0.1%)		17 (0.6%)	13 (0.7%)	2 (0.3%)		
Hepato-gastro-enterology (chronic)	1616 (11.2%)	791 (10.9%)	523 (11.3%)		327 (11.0%)	199 (10.9%)	68 (10.6%)		
Hepato-gastro-enterology (infectious)	1152 (8.0%)	485 (6.6%)	448 (9.7%)		124 (4.2%)	69 (3.8%)	33 (5.2%)		
Infectiology	1921 (13.3%)	922 (12.7%)	665 (14.4%)		545 (18.3%)	311 (17.0%)	128 (20.0%)		
Medical care	265 (1.8%)	163 (2.2%)	63 (1.4%)		42 (1.4%)	29 (1.6%)	10 (1.6%)		
Neurology	984 (6.8%)	638 (8.8%)	141 (3.1%)		222 (7.5%)	155 (8.5%)	36 (5.6%)		
Non-specific diagnostics	805 (5.6%)	466 (6.4%)	189 (4.1%)		269 (9.1%)	195 (10.7%)	33 (5.2%)		
Non-traumatic rheumatology	653 (4.5%)	334 (4.6%)	189 (4.1%)		178 (6.0%)	124 (6.8%)	26 (4.1%)		
Non-traumatology ophthalmology	132 (0.9%)	46 (0.6%)	67 (1.5%)		24 (0.8%)	11 (0.6%)	11 (1.7%)		
Pneumology (chronic)	424 (2.9%)	292 (4.0%)	75 (1.6%)		115 (3.9%)	85 (4.7%)	12 (1.9%)		
Pneumology (infectious)	783 (5.4%)	373 (5.1%)	268 (5.8%)		61 (2.1%)	39 (2.1%)	17 (2.7%)		
Poisoning and addiction	61 (0.4%)	27 (0.4%)	16 (0.4%)		12 (0.4%)	6 (0.3%)	2 (0.3%)		
Psychiatry and psychological problems	408 (2.8%)	236 (3.2%)	90 (1.9%)		135 (4.5%)	72 (4.0%)	26 (4.1%)		
Social, administrative, and forensic problems	314 (2.2%)	118 (1.6%)	149 (3.2%)		63 (2.1%)	27 (1.5%)	25 (3.9%)		
Urogenital	474 (3.3%)	241 (3.3%)	155 (3.4%)		99 (3.3%)	52 (2.9%)	24 (3.7%)		
**Pathology**				<0.001				<0.001	
Chronic diseases	6177 (42.7%)	3422 (47.0%)	1603 (34.6%)		1476 (49.6%)	953 (52.2%)	270 (42.1%)		
Infectious diseases	5730 (39.6%)	2595 (35.6%)	2150 (46.4%)		902 (30.3%)	502 (27.5%)	239 (37.3%)		
Others	2562 (17.7%)	1264 (17.4%)	876 (18.9%)		595 (20.0%)	370 (20.3%)	132 (20.6%)		

SD – standard deviation

*P-value of comparisons between men and women; the mapped ICD10 to chronic disease is included in the **Online Supplementary Document**.

**Figure 2:**
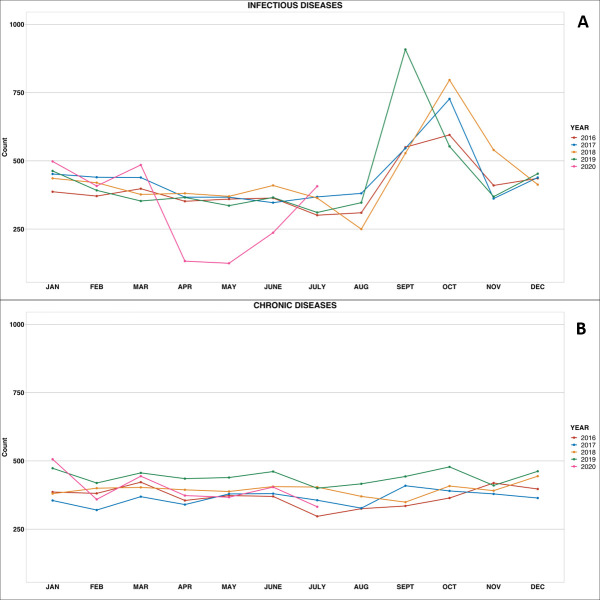
Broad diagnostic categories of emergency medical consultations over five years. **Panel A.** Infectious diseases. **Panel B.** Chronic diseases.

We first examined all consultations in the overall population grouped under infectious and chronic consultations, categorized per month over five years. The trend pattern in the primary cause of consultations between January and July remains similar between 2016 and 2019 (**Figure 2**, Panels A and B, respectively). In 2020, the aggregate data showed a drop in the number of cases of infectious diseases from April (starting date of the application of COVID-19 prevention measures, especially quarantines measures) to May, as compared to the same period in the last five years (**Figure 2**, Panel A) (366.5 and 358.2 in April and May 2016-2019 compared to 133.0 and 125.0 in 2020). In contrast, the corresponding prevalence of chronic conditions remained stable over the study period (**Figure 2**, Panel B) (381.0 and 394.7 in April and May 2016-2019 compared to 373.0 and 367.0 in 2020). We observed a similar and consistent pattern across age groups and in men and women (Supplemental Figure 2 and Supplemental Figure 3 in the **Online Supplementary Document**). In a stratified analysis by subtype of infectious and chronic diseases (Supplemental Figure 4 in the **Online Supplementary Document**), the data showed a sharp decline in consultations for infections consultations particularly for ENT-Stomatology while non-communicable diseases (NCDs) remained stable, except for psychiatry consultations that rose from April to May 2020 compared to previous years.

**Figure 3:**
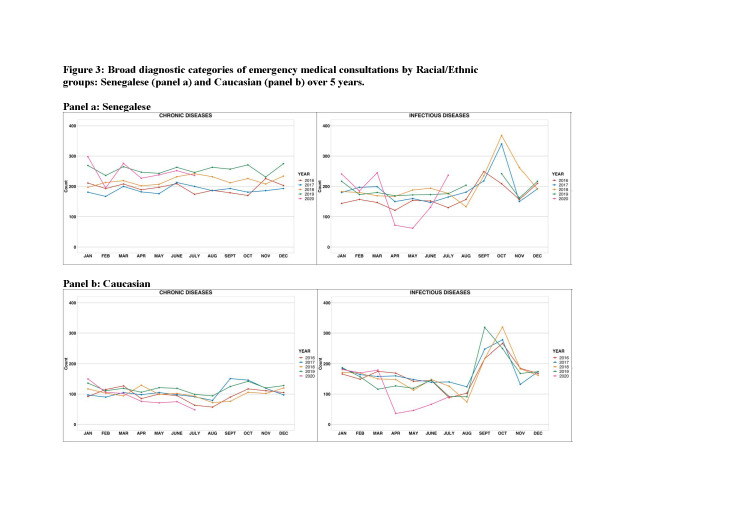
Broad diagnostic categories of emergency medical consultations by racial/ethnic groups over five years. **Panel A.** Senegalese. **Panel B.** Caucasian.

We then examined consultations between April and July in the overall Dakar population stratified by ethnicity group. The pattern of trend in the primary cause of consultations between April and July was similar across both ethnic groups between 2016 and 2020 (**Table 1** and **Figure 3**, Panels A and B, respectively). Supplemental Table 1 in the **Online Supplementary Document** presents a comparison of the characteristics of the consultations from April-May 2020 with June-July 2020 in Senegalese vs Caucasian patients.

In the multivariable logistic regression analysis after adjustment for age and sex, **C**onsultations for infectious diseases were significantly more likely to occur from April and May 2016 to 2019 as compared to the same period in 2020 ((2016 OR = 2.4; 95% CI = 2.0 to 2.9), (2017 OR = 2.7; 95% CI = 2.3-3.4), (2018 OR = 2.4; 95% CI = 2.0-2.9) and (2019 OR = 2.0; 95% CI = 1.7-2.5)) (**Table 2**).

**Table 2:** Multivariable ORs for excess risk of infectious diagnosis in April-May 2020 as compared to the same 2016-2019 period

	OR (95% CI)*	*P*-value
2016	2.4 (2.0-2.9)	<0.001
2017	2.7 (2.3-3.4)	<0.001
2018	2.4 (2.0-2.9)	<0.001
2019	2.0 (1.7-2.5)	<0.001
Sex (men)	1.1 (1.0-1.3)	<0.05
Age = 20-45 years†	0.4 (0.3-0.5)	<0.001
Age ≥45 years	0.2 (0.1-0.3)	<0.001

OR – odds ratio, CI – confidence interval

*Logistic regression adjusted for age and sex, with n = 5721 and 2020 as the reference exposure category.

†Reference age is <20 years.

After adjusting for age and sex, there was a significantly increased rate of consultation for infectious diagnosis in June and July as compared to April and May 2020 (**Table 3**).

**Table 3:** Multivariable ORs for excess risk of infectious diagnosis in June and July as compared to April-May 2020

	OR (95% CI)*	*P*-value
June/July	2.6 (2.1-3.1)	<0.0001
Sex (men)	1.7 (1.4-2.1)	<0.0001
Age = 20-45 years†	0.6 (0.5-0.8)	<0.001
Age ≥45 years	0.4 (0.3-0.5)	<0.0001

OR – odds ratio, CI – confidence interval

*Logistic regression adjusted for age and sex, with n = 1964 and April-May as the reference exposure category.

†Reference age is <20 years.

## DISCUSSION

We reported two key findings based on data from consultations in a large, well-documented medical emergency medical service in Dakar. First, we observed a decrease in consultation for infectious diseases starting from the application of COVID-19 sanitary measures (April-May 2020) as compared to the same period over the previous five years, while the number of consultations for chronic diseases remained stable. Second, in our study, we do not report any disparities across ethnic groups in terms of the number of consultations for both infectious and chronic diseases.

During the period covering the implementation of COVID-19 sanitary measures (April to May 2020), infectious disease consultations dropped significantly, while chronic diseases remained stable. During an outbreak such as the COVID-19 pandemic, consultations for infectious disease rates would presumably increase as the virus spreads throughout the country. However, as previously reported, COVID-19 has not affected African countries as dramatically as western ones [4]. The decrease in infectious disease cases we found is a first step toward understanding the low spread of the COVID-19 disease in Africa. Our finding suggests that sanitary measures may have limited the spread of COVID-19 and other infectious diseases.

Each country implemented swift preventative measures that contributed to controlling the transmission of the virus. Such prevention methods included border control, travel restriction, the use of face coverings/masks, social distancing (maintaining a distance of 6 feet from others), social media campaigns, quarantines, and emphasis on proper sanitation and hygiene practices [8]. A continent-wide response, the Africa Taskforce on Coronavirus Preparedness and Response (AFTCOR), was also carried out [9]. The AFTCOR was formed by the African Center for Disease Control and Prevention (Africa CDC) and the Southern Africa Center for Infectious Disease Surveillance (SACIDS) [10]. Such centralized response included surveillance in high-risk countries and provided proper laboratory testing and diagnosis; both strategies were deemed successful during the 2014 and 2018 Ebola epidemics [4,10].

Notably, infectious disease cases began to rise again between May and July, which could be explained by the sudden increase could be that sanitary measures were no longer enforced as strictly as they once were at the beginning of the outbreak. The loosened COVID-19 restrictions have led people to take fewer precautions, resulting in increased transmission of infectious diseases, including COVID-19. Furthermore, this finding carries large public health implications, as the strategies used to limit the spread of COVID-19 can be used to combat any other potential epidemics that plague sub-Saharan Africa, particularly Ebola, tuberculosis, or diarrheal diseases.

Another possible contribution to the decline in infectious disease cases is people’s possible hesitancy to visit health facilities because of the fear of catching the virus, especially if they were experiencing a md disease. Chronic diseases such as cardiovascular diseases (CVDs) and other NCDs are more severe at times and require continuous monitoring, possibly explaining why chronic disease consultations did not decrease when sanitary measures were applied in Senegal. In contrast, many consultations and appointments were postponed due to COVID-19 in Europe [11]. The UK is predicted to have an estimated increase of about 18 000 deaths annually due to delayed diagnosis and treatment of cancer patients during the pandemic. while in France, 38% of patients reported cancelling medical services due to the fear of infection and 28% due to fear of disturbing physicians during a pandemic [11].

Surprisingly, our results show that consultations for chronic diseases during the COVID-19 outbreak remained stable compared to the previous five years. Evidence shows that social isolation is expected to negatively affect a population’s mental and physical health, especially during an outbreak such as this one. The COVID-19 sanitary measures required people to stay inside their homes, leaving many unable to work or go to school. We observed a higher rate of consultation for psychiatry during the first months of implementation of COVID-19 hygienic measures. Although research on the effects of sanitary measures on mental health during COVID-19 is limited, studies on previous epidemics have shown an increase in psychiatric symptoms [12] and cardiac conditions [13] during and after control measures were implemented. A study on the Ebola outbreak of 2014 showed the prevalence of 48% for anxiety and depression and 76% for posttraumatic stress disorder (PTSD) due to the Ebola response; similar findings were identified during the severe acute respiratory syndrome (SARS) and H1N1 pandemic influenza outbreaks [12].

Our study did not highlight disparities in morbidity or mortality during the COVID-19 period across racial/ethnic, unlike the studies in the USA and the UK [14]. Blacks and other minority groups faced a higher rate of COVID-19 cases and deaths in the US and the UK, as well as an overall increased burden of chronic diseases during the pandemic [6,15]. Rao et al. in the USA demonstrated that Black patients hospitalized with severe COVID-19 between March and July 2020 were at increased odds of requiring mechanical ventilation compared to White patients, but not in mortality [16]. Also, a systematic review of 52 studies by Khanijahani et al. highlighted that Black people were at higher risk of COVID-19 infections, while studies were not consistent regarding mortality [17]. Several causes have been posited for the disparities, including social determinants of health, pre-existing conditions, genetics, structural racism, access to health care, and behavioral and economic aspects [4]. We found that Senegalese and Caucasian patients are likely to have quite similar socioeconomic status, which favors socioeconomic status playing a major role in the differences observed between ethnic groups in the US and the UK. Moreover, Senegal has tackled COVID-19 aggressively, which has so far led to a very low number of COVID-19 cases and deaths. More than six months into the pandemic, the country has about 14 000 cases and 284 deaths, making comparisons at the ethnic level challenging given the lack of statistical power.

Overall, this study represents unique data on the immediate effect of sanitary measures during COVID-19 in Senegal and provides relevant information that will help to pursue further research in Senegal and other Sub-Saharan African countries.

This study has some limitations. First, it only addresses emergency consultations. Furthermore, we used a single database, SOS Médecins Dakar, to address our questions, so it is unclear whether our observations reflect a direct association between COVID-19 prevention measures and their effect on consultations. Additionally, although we based this study on the largest database of emergency medical consultations in Senegal, the extent to which it is fully representative of health care utilization in emergency medicine in an urban setting is unclear, as other emergency care services were not involved due to lack of documentation. We derived data from the capital city, so it is possible that disease patterns and resource utilization in other regions, especially more rural areas, may be different. Although our database had few missing data on the patient’s sex, age, ethnicity, and diagnosis for the consultation, there might be a differential selection bias for excluded patients. Also, data collection conditions may have been impacted by the pandemic. However, this study provides unique data on the potential effect of sanitary measures against COVID-19 in Africa and emphasizes the need to limit the spread of all infectious diseases in this part of the world where they are still highly prevalent.

## CONCLUSIONS

African countries should keep implementing sanitary measures, including the use of face masks, social distancing, hand cleaning, and remaining vigilant in the upcoming weeks or months. Furthermore, the absence of disparities across ethnic groups can further guide research on the mechanism underlying the COVID-19 racial gap found in the UK and the US.

## Additional material


Online Supplementary Document
