# Supplementary material for: Time trends in infectious and chronic disease consultations in Dakar, Senegal: Impact of COVID-19 sanitary measures
Source: J Glob Health. 2023 Mar 31;13:06007. doi: 10.7189/jogh.13.06007 (PMC10062398; doi:10.7189/jogh.13.06007)
Supplement: Online Supplementary Document [file jogh-13-06007-s001.pdf]

Supplemental Data

- **e-Figure 1.** Flowchart of Sample Selection.
- **e-Figure 2.** Number of emergency medical consultations for chronic and infectious diseases by age group (panel a) and gender (panel b) over a 5-year period.
- **e-Figure 3.** Broad diagnostic categories of emergency medical consultations for chronic and infectious diseases by age group (Panel a) and gender (Panel b) over a 5-year period.
- **e-Figure 4.** Number of emergency medical consultations for Cardiovascular (Panel a), Non-Traumatic Rheumatology (Panel b), Psychiatry and Psychological (Panel c), and Ear Nose and Throat and Stomatology (Panel d) disease over a 5-year period
- **e-Table 1.** Characteristics of the consultations from April to May (2020) as compared to June to July (2020).

e-Figure 1: Flow chart of Sample Selection

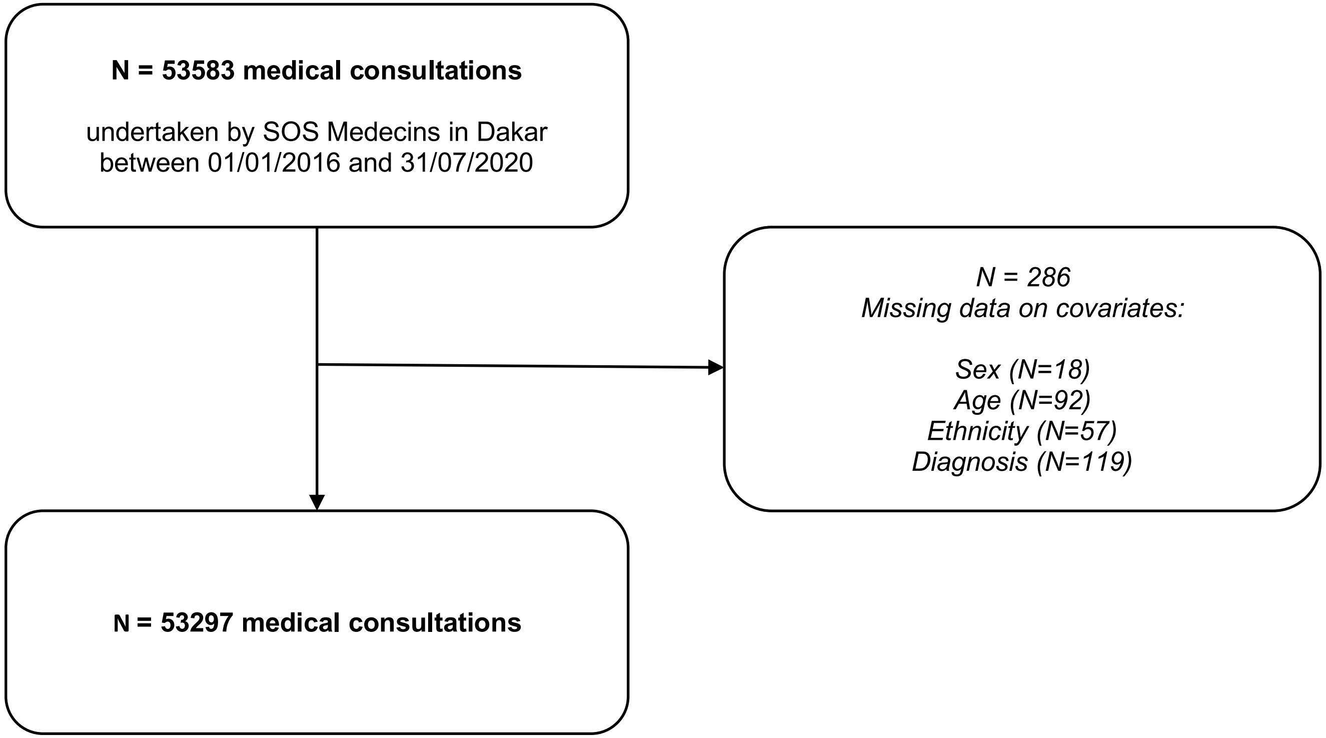

e-Figure 2: Number of emergency medical consultations for chronic and infectious diseases by age group (panel a) and gender (panel b) over a 5-year period.

Panel a

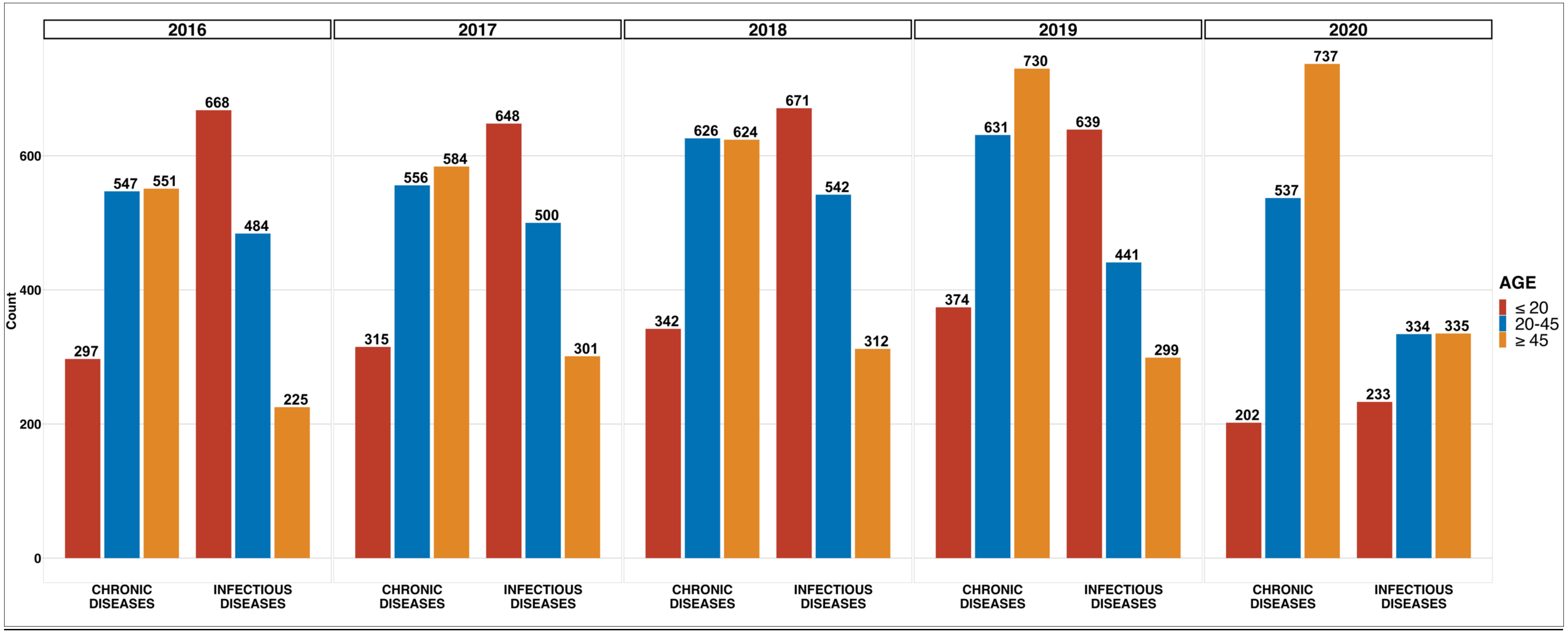

Panel b

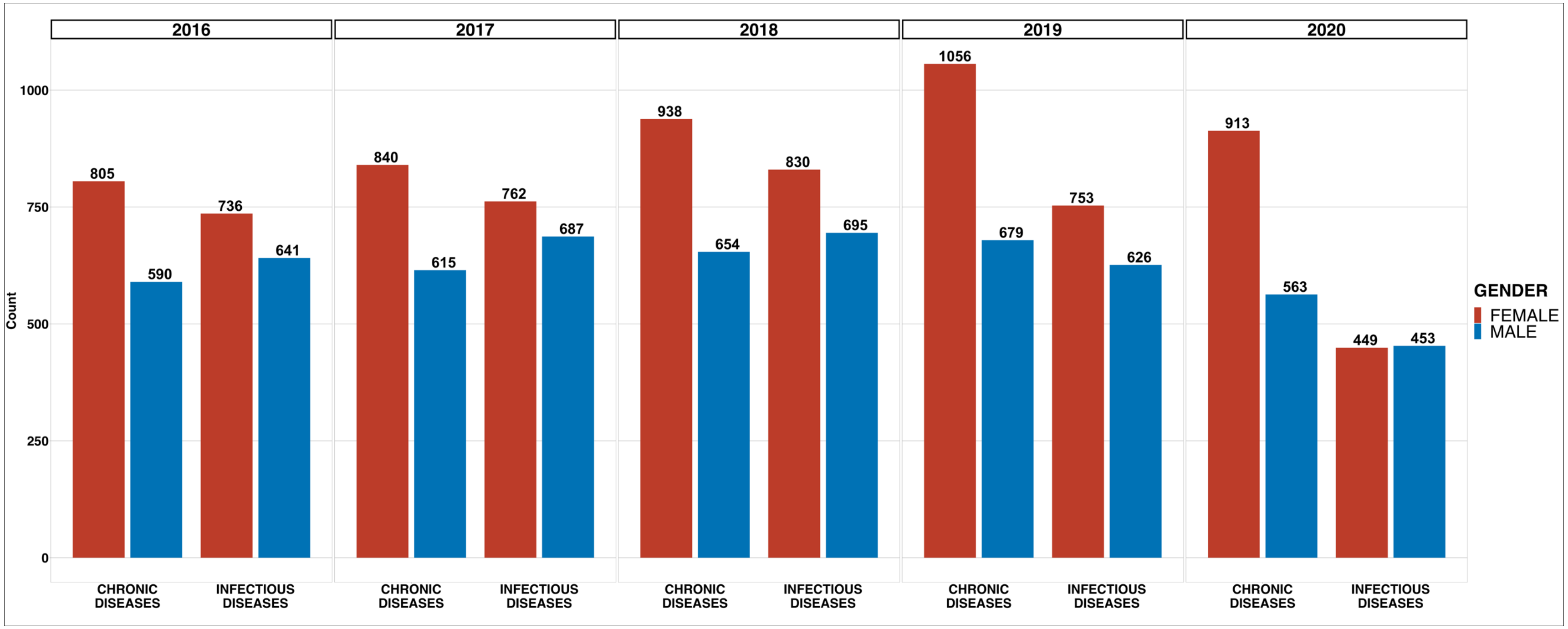

2016
2017
2018
2019
2020
Count
1000
750
500
250
0
805
590
736
641
840
615
762
687
938
654
830
695
1056
679
753
626
913
563
449
453
CHRONIC DISEASES
INFECTIOUS DISEASES
GENDER
FEMALE
MALE

e-Figure 3: Broad diagnostic categories of emergency medical consultations for chronic and infectious diseases by age group (Panel a) and gender (Panel b) over a 5-year period.

Panel a

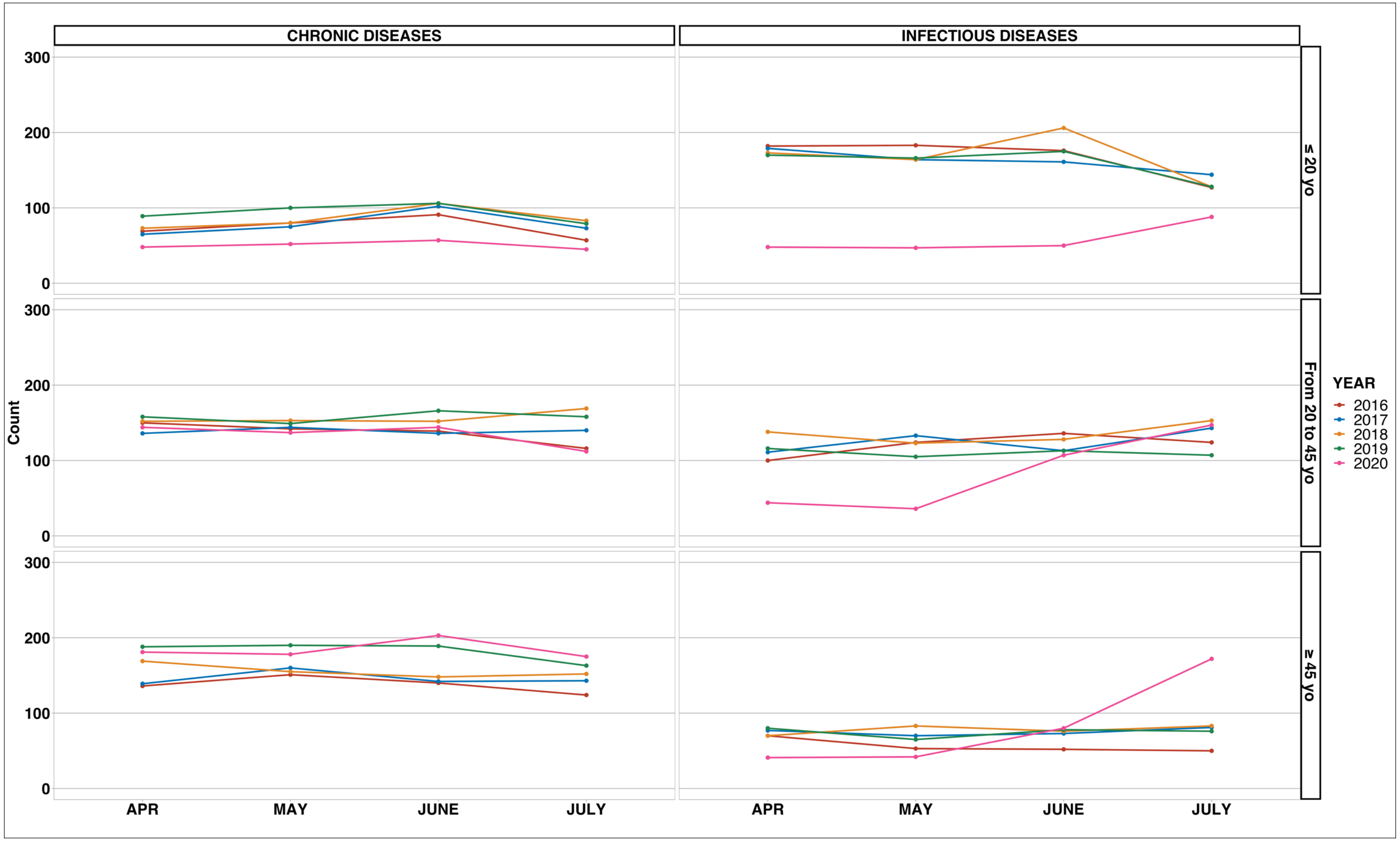

Panel b

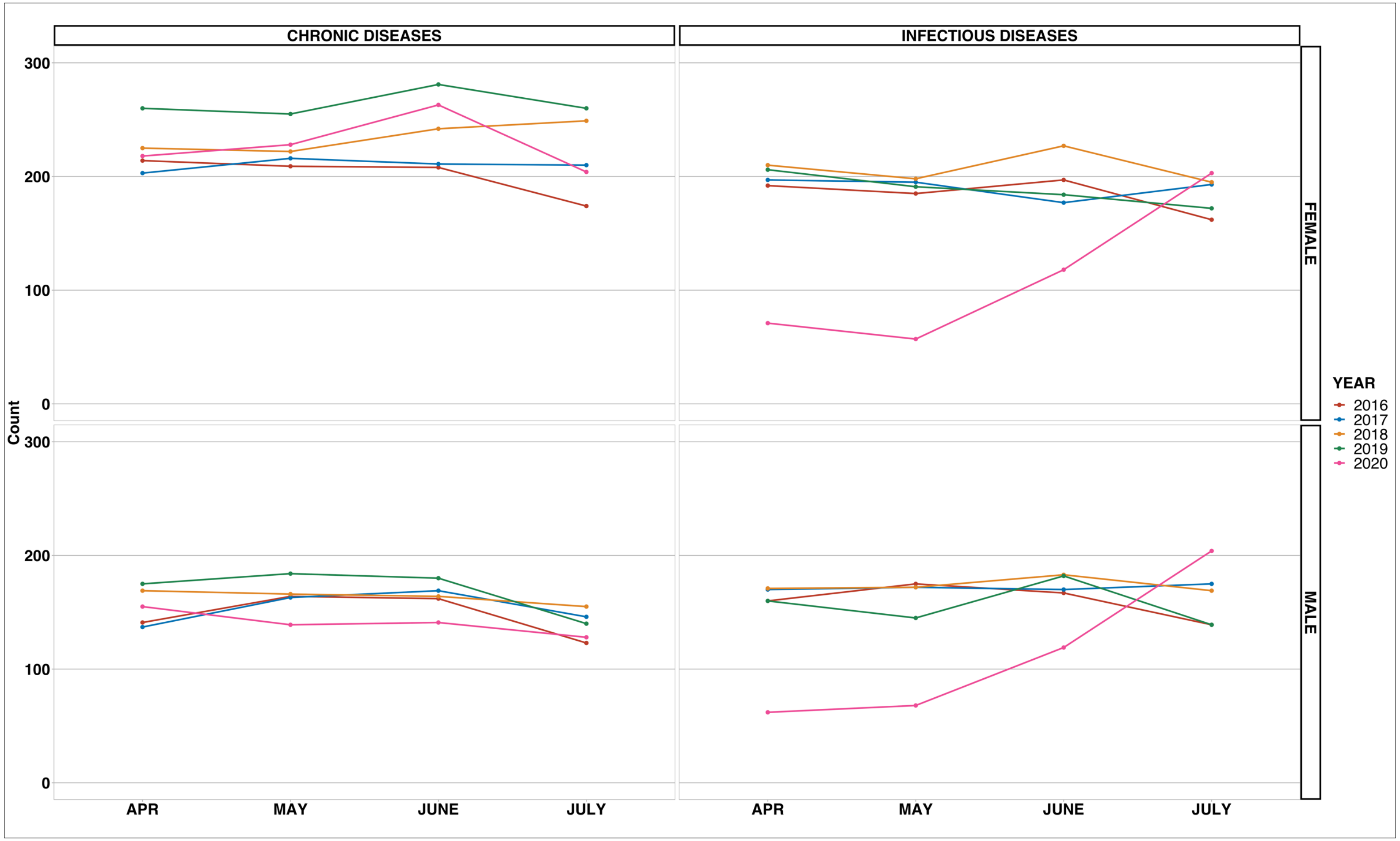

CHRONIC DISEASES
INFECTIOUS DISEASES
FEMALE
MALE
Count
300
200
100
0
APR
MAY
JUNE
JULY
YEAR
2016
2017
2018
2019
2020

e-Figure 4: Broad diagnostic categories of emergency medical consultations by Cardiovascular (Panel a), Non-Traumatic Rheumatology (Panel b), Psychiatry and Psychological conditions (Panel c), and ENT and infectious Stomatology (Panel d) disease over a 5-year period

Panel a

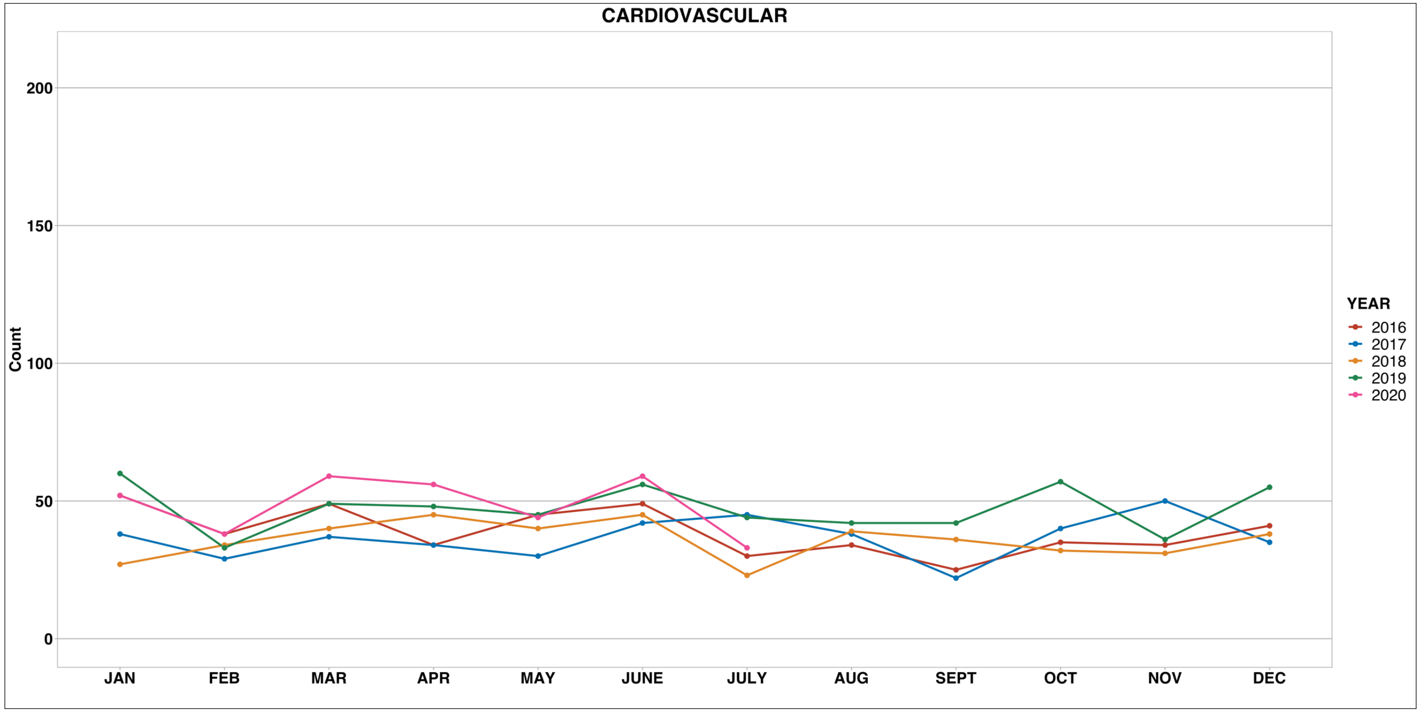

Panel b

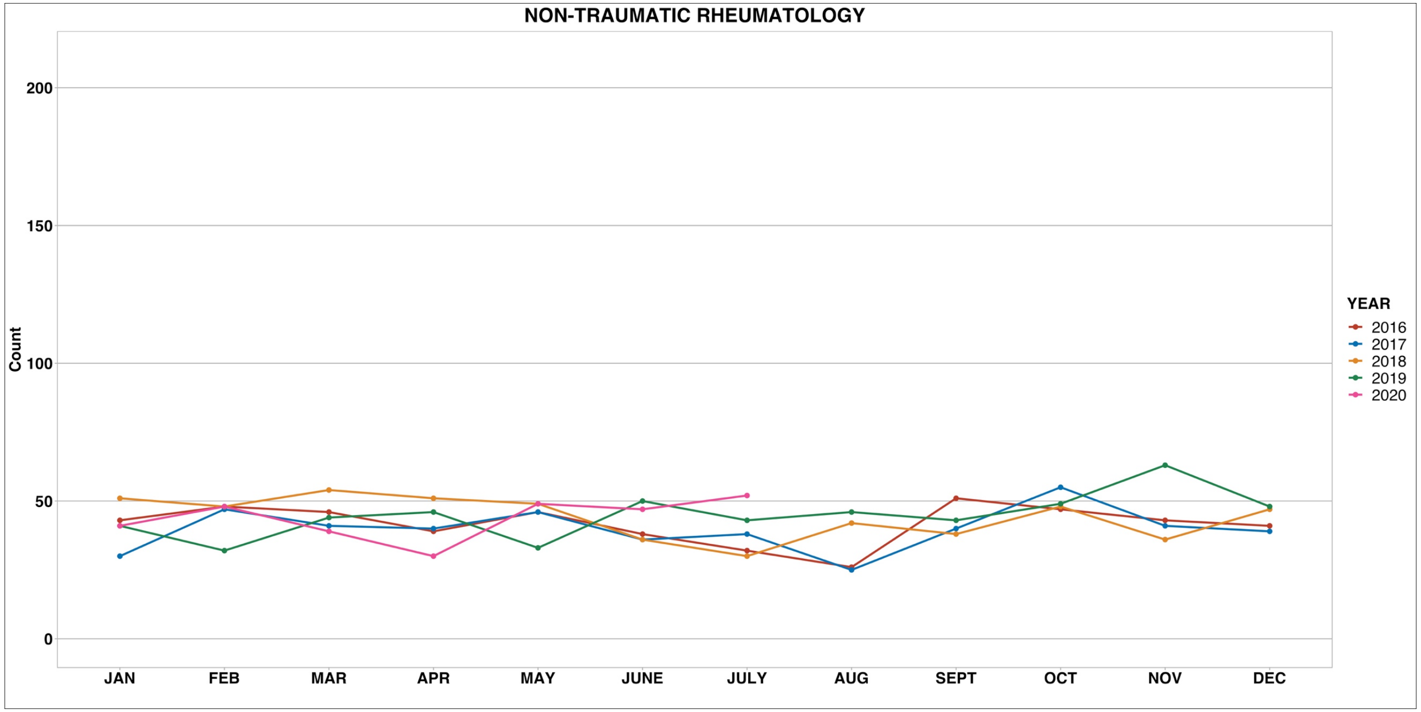
NON-TRAUMATIC RHEUMATOLOGY
Count
0
50
100
150
200
JAN
FEB
MAR
APR
MAY
JUNE
JULY
AUG
SEPT
OCT
NOV
DEC
YEAR
2016
2017
2018
2019
2020

Panel c

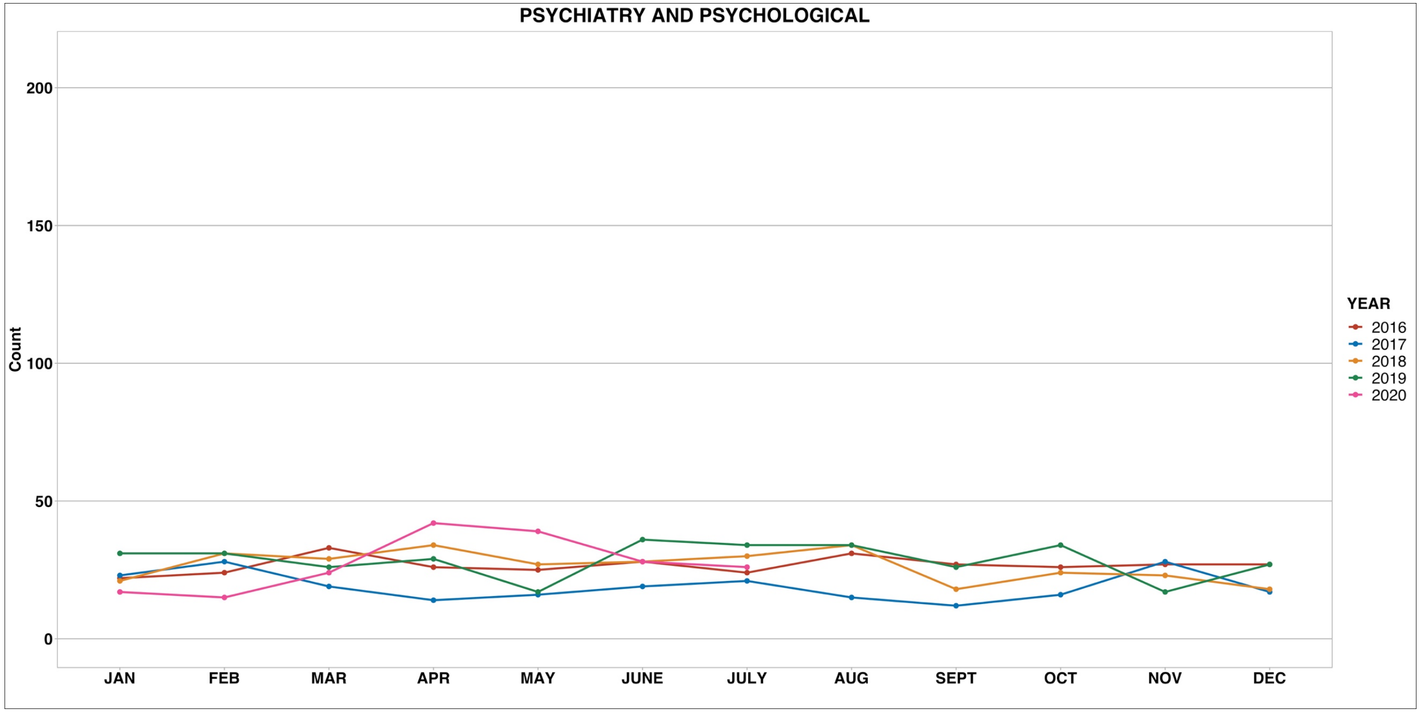
PSYCHIATRY AND PSYCHOLOGICAL
Count
0
50
100
150
200
JAN
FEB
MAR
APR
MAY
JUNE
JULY
AUG
SEPT
OCT
NOV
DEC
YEAR
2016
2017
2018
2019
2020

Panel d

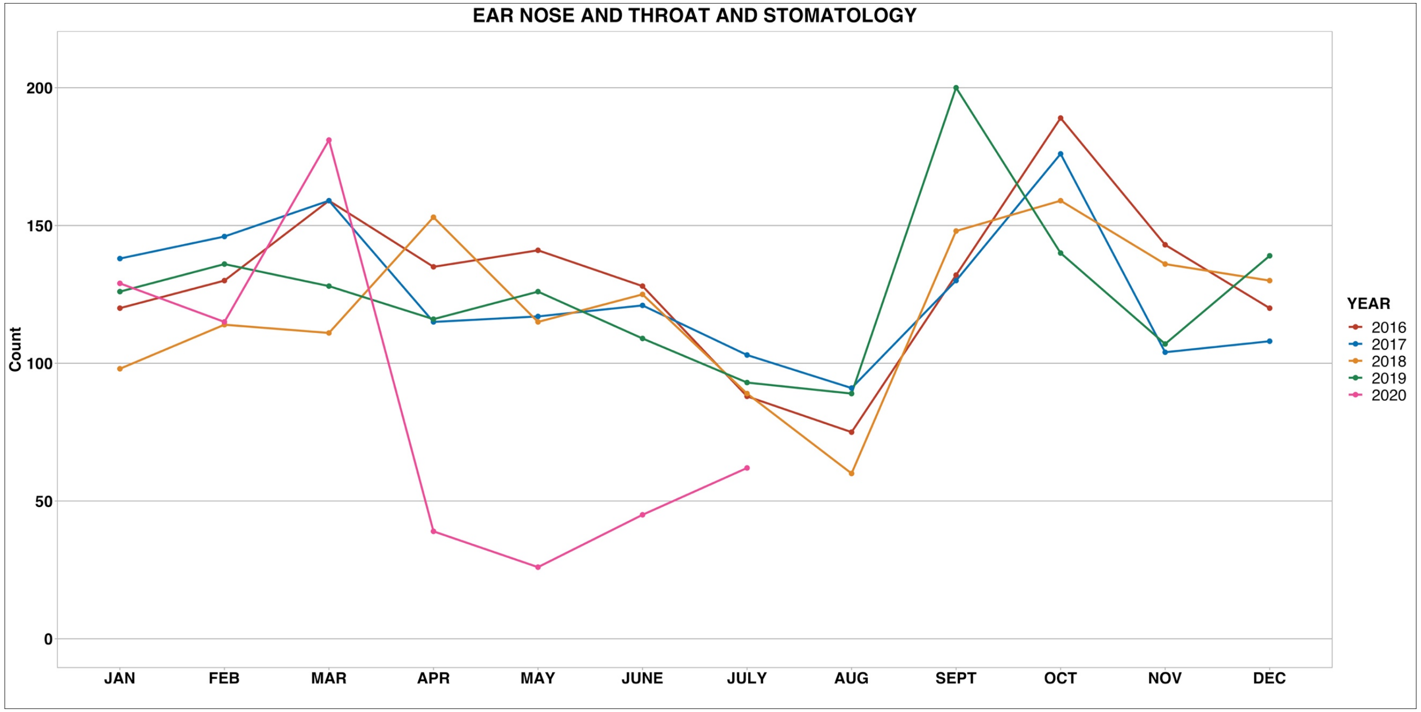

EAR NOSE AND THROAT AND STOMATOLOGY
Count
0
50
100
150
200
JAN
FEB
MAR
APR
MAY
JUNE
JULY
AUG
SEPT
OCT
NOV
DEC
YEAR
2016
2017
2018
2019
2020

e-Table 1. Characteristics of the consultations from April to May (2020) as compared to June to July (2020).

	From April to May 2020				From June to July 2020			
	[ALL] *N=1270*	SENEGALESE *N=761*	CAUCASIAN *N=305*	p.overall	[ALL] *N=1703*	SENEGALESE *N=1064*	CAUCASIAN *N=336*	p.overall
AGE	42.5 (24.2)	43.9 (24.8)	39.1 (24.2)	0.004	45.1 (24.2)	46.6 (24.5)	39.9 (22.4)	<0.001
AGE_2:				<0.001				0.013
0_≤20	249 (19.6%)	138 (18.1%)	86 (28.2%)		285 (16.7%)	164 (15.4%)	72 (21.4%)	
1_>20	1021 (80.4%)	623 (81.9%)	219 (71.8%)		1418 (83.3%)	900 (84.6%)	264 (78.6%)	
AGE_3:				0.001				0.024
0_≤20	249 (19.6%)	138 (18.1%)	86 (28.2%)		285 (16.7%)	164 (15.4%)	72 (21.4%)	
1_20-45	443 (34.9%)	266 (35.0%)	85 (27.9%)		601 (35.3%)	376 (35.3%)	119 (35.4%)	
2_≥45	578 (45.5%)	357 (46.9%)	134 (43.9%)		817 (48.0%)	524 (49.2%)	145 (43.2%)	
SEXE:				0.022				0.005
F	720 (56.7%)	452 (59.4%)	157 (51.5%)		955 (56.1%)	623 (58.6%)	167 (49.7%)	
M	550 (43.3%)	309 (40.6%)	148 (48.5%)		748 (43.9%)	441 (41.4%)	169 (50.3%)	
PATHOLOGIE_3:				.				.
ALL TRAUMA	100 (7.87%)	58 (7.62%)	25 (8.20%)		85 (4.99%)	44 (4.14%)	26 (7.74%)	
CARDIOVASCULAR	100 (7.87%)	76 (9.99%)	15 (4.92%)		92 (5.40%)	67 (6.30%)	8 (2.38%)	
DERMATOLOGY	29 (2.28%)	8 (1.05%)	18 (5.90%)		27 (1.59%)	11 (1.03%)	12 (3.57%)	
ENDOCRINOLOGY, METABOLIC TR	18 (1.42%)	16 (2.10%)	1 (0.33%)		28 (1.64%)	19 (1.79%)	5 (1.49%)	
ENT AND STOMATOLOGY (CHRONIC)	51 (4.02%)	37 (4.86%)	6 (1.97%)		38 (2.23%)	19 (1.79%)	11 (3.27%)	
ENT AND STOMATOLOGY (INFECTIOUS)	65 (5.12%)	36 (4.73%)	19 (6.23%)		107 (6.28%)	47 (4.42%)	42 (12.5%)	
HEMATO-ONCOLOGY	6 (0.47%)	6 (0.79%)	0 (0.00%)		11 (0.65%)	7 (0.66%)	2 (0.60%)	
HEPATO-GASTRO-ENTEROLOGY (CHRONIC)	171 (13.5%)	100 (13.1%)	38 (12.5%)		156 (9.16%)	99 (9.30%)	30 (8.93%)	
HEPATO-GASTRO-ENTEROLOGY (INFECTIOUS)	57 (4.49%)	30 (3.94%)	17 (5.57%)		67 (3.93%)	39 (3.67%)	16 (4.76%)	
INFECTIOLOGY	117 (9.21%)	57 (7.49%)	39 (12.8%)		428 (25.1%)	254 (23.9%)	89 (26.5%)	
MEDICAL CARE	19 (1.50%)	11 (1.45%)	7 (2.30%)		23 (1.35%)	18 (1.69%)	3 (0.89%)	
NEUROLOGY	113 (8.90%)	74 (9.72%)	21 (6.89%)		109 (6.40%)	81 (7.61%)	15 (4.46%)	
NON-SPECIFIC DIAGNOSTICS	109 (8.58%)	75 (9.86%)	21 (6.89%)		160 (9.40%)	120 (11.3%)	12 (3.57%)	
NON-TRAUMATIC RHUMATOLOGY	79 (6.22%)	54 (7.10%)	11 (3.61%)		99 (5.81%)	70 (6.58%)	15 (4.46%)	
NON-TRAUMATOLOGY OPHTALMOLOGY	14 (1.10%)	7 (0.92%)	6 (1.97%)		10 (0.59%)	4 (0.38%)	5 (1.49%)	

PNEUMOLOGY (CHRONIC)	34 (2.68%)	28 (3.68%)	2 (0.66%)		81 (4.76%)	57 (5.36%)	10 (2.98%)	
PNEUMOLOGY (INFECTIOUS)	19 (1.50%)	11 (1.45%)	7 (2.30%)		42 (2.47%)	28 (2.63%)	10 (2.98%)	
POISONING AND ADDICTION	6 (0.47%)	2 (0.26%)	2 (0.66%)		6 (0.35%)	4 (0.38%)	0 (0.00%)	
PSYCHIATRY AND PSYCHOLOGICAL PB	81 (6.38%)	41 (5.39%)	18 (5.90%)		54 (3.17%)	31 (2.91%)	8 (2.38%)	
SOCIAL, ADM., FORENSIC PB	24 (1.89%)	9 (1.18%)	15 (4.92%)		39 (2.29%)	18 (1.69%)	10 (2.98%)	
UROGENITAL	58 (4.57%)	25 (3.29%)	17 (5.57%)		41 (2.41%)	27 (2.54%)	7 (2.08%)	
PATHOLOGIE_4:				<0.001				<0.001
CHRONIC DISEASES	740 (58.3%)	465 (61.1%)	147 (48.2%)		736 (43.2%)	488 (45.9%)	123 (36.6%)	
INFECTIOUS DISEASES	258 (20.3%)	134 (17.6%)	82 (26.9%)		644 (37.8%)	368 (34.6%)	157 (46.7%)	
OTHERS	272 (21.4%)	162 (21.3%)	76 (24.9%)		323 (19.0%)	208 (19.5%)	56 (16.7%)	
